# Inside the Syntactic Box: The Neural Correlates of the Functional and Positional Level in Covert Sentence Production

**DOI:** 10.1371/journal.pone.0106122

**Published:** 2014-09-30

**Authors:** Simona Collina, Ruth Seurinck, Robert J. Hartsuiker

**Affiliations:** 1 Università degli Studi Suor Orsola Benincasa, Naples, Italy; 2 Department of Data-Analysis and Ghent Institute for Functional and Metabolic Imaging, Ghent University, Ghent, Belgium; 3 Department of Experimental Psychology, Ghent University, Ghent, Belgium; University of Milan, Italy

## Abstract

The aim of the present fMRI study was to investigate the neural circuits of two stages of grammatical encoding in sentence production. Participants covertly produced sentences on the basis of three words (one verb and two nouns). In the *functional level* condition both nouns were animate and so were potential competitors for the grammatical function of subject. In the *positional level* condition the first noun was animate whereas the second was inanimate. We found activation of Broca's and adjacent areas, previously indicated as responsible for syntactic processing. Additionally, a later onset of the activation in three brain areas in the functional level condition suggests that there is indeed a competition for assignment of subjecthood. The results constrain theories of grammatical encoding, which differ in whether they assume two separate processing levels or only one.

## Introduction

The aim of this study was to investigate the neural circuits involved in grammatical encoding, that is, the processing level that specifies the structure of an utterance. This level plays its part after the creation of a message to convey the intended meaning and before the retrieval of the phonological features of a sentence, and consists of a number of processes that realize the intended concepts into a linguistic plan ([1,2]). The psycholinguistic model of sentence production proposed by Bock and Levelt [1] can be considered one of the most exhaustive theoretical descriptions of the stages to be achieved by a speaker in order to correctly produce a sentence. In the model, four different levels of processing are postulated. First, there is a message level in which the conceptual knowledge to be expressed is generated. At this stage, the system uses the knowledge of the world a speaker has. Second, there is a functional level in which words with their semantic and syntactic properties are retrieved from the lexicon to express the intended meaning (lexical selection) and where the syntactic role of each word is assigned (function assignment). For example, a speaker may intend to produce “*the cat chases the ball*”. Words that must be selected and retrieved from the mental lexicon are two nouns (*cat, ball*) and a verb (*to chase*). Then the syntactic functions are assigned, namely the subject function (*the cat*), the object function (*the ball*) and the verb function (*to chase*). At this level, the functions of the words are defined but their order is not. Third, there is the positional level during which a hierarchical constituent structure is established allowing the speaker to put the elements in the correct order (constituent assembly) and during which the morphological processes (inflection) take place. This level determines that, for example, *the cat* is placed at the beginning of the sentence and that the verb *to chase* carries the right morphological markers for third person singular present. So it is at this level that the previously specified functions are combined with information about, for example, the tense, the aspect and the number. Finally, the fourth level of phonological encoding level retrieves and assembles the sounds of the sentence before articulation [3].

The two-stage model of grammatical encoding (i.e., the distinction between a functional and a positional level) is supported by various sources of evidence. For instance, important support for the model comes from speech error analyses. Semantic substitutions as in *hot under the belt*, when *collar* is intended [4] and word exchange errors as *my boy terrifies the cat next door* when *my cat terrifies the boy next door* is intended [5], [6], provide evidence for a level at which grammatical class constraints and roles are set. In contrast, stranding errors demonstrate that there is a level where serial order is decided [6]. As noted by Garrett [7] in the sentence *I went to get my park trucked* the words *truck* and *park* switch position, but the suffix -*ed* is in the correct location suggesting that inflectional morphemes are part of the phrasal frame. An experiment investigating subject-verb agreement revealed that agreement relations are processed before word order determination takes place [8]. Finally, experiments on syntactic priming effects (the tendency to produce a sentence with a syntactic structure because of a previously heard sentence with the same structure) demonstrated that word order can be primed [9], which is consistent with a separate level devoted to word order computation.

The two-stage model is also consistent with studies that tested the effects of conceptual and lexical variables on speaker's assignment of subjecthood to nouns. McDonald, Bock, Kelly [10] found that animate nouns tend to be used as subjects of sentences. Participants heard and later tried to remember sentences or phrases that contained two target nouns. The target nouns differed for animacy and a number of other variables as, for example, number of syllables and stress patterns. Results evidenced that animate nouns tended to be produced before inanimate in transitive sentences. As suggested by Comrie [11] the most natural transitive construction has a noun in subject position which is “prominent” that is, high in features as, for example, animacy and definiteness. Along these lines, Bock and Warren [12] found that concrete nouns tend to be used as subjects. Bock and colleagues concluded that the results are a consequence of the assignment to grammatical functions (functional level) rather than the product of a linearization process (positional level). Words that are conceptually more accessible because of their meaning, their concreteness value, or because they are animate, tend to be assigned to higher roles in grammatical relation hierarchies (subject dominates direct object and direct object dominates indirect object) [13] when grammatical functions processes take place. Similar conclusions were reached also by Bornkessel-Schlesewsky and Schlesewsky in sentence comprehension studies [14]. An important consequence for our purposes is that two animate entities compete to be assigned to grammatical functions (subject, direct object). Such competition is supported by several further studies. Specifically, Smith and Wheeldon [15] observed that participants were slower describing a picture by producing an utterance when two nouns were conceptually similar. Further, Meyer [16] presented her participants with pairs of objects and required them to produce either a noun phrase conjunction “*the arrow and the bag*” or a short sentence as “*the arrow is next to the bag*”. Semantically or phonologically related distracter words accompanied the stimuli presentation. Onset naming latencies were longer in presence of a distracter that was semantically related to either of the two nouns, whereas facilitation occurred with a phonological related distracter only for the first noun. The results suggested that before producing such an utterance, both target lemmas need to be selected. When two thematic roles (agent and theme) are equally accessible because both are animate (cat, dog), they compete for the grammatical assignment to the role of subject. On the contrary, when the agent is animate and the theme is inanimate (cat, ball), no competition occurs for the assignment in the subject position.

The study of neural circuits involved in grammatical encoding has not received the same level of attention as behavioural studies. Because of the difficulty to control variables involved in the production of utterances, especially in the experimental setting of fMRI, many authors concentrated on the study of the neural circuits involved in single word processing, providing evidence about how words are lexically organized, represented and retrieved [17]. Importantly, although there are a few studies that have investigated the neural circuits of sentence production, none of them have made a distinction between functional processes (assignment of subject, object, …) and positional processes (word order determination). This is surprising given the solid theoretical basis and large base of evidence from behavioural studies for such a distinction, as briefly reviewed above. To begin to fill in this gap, the present study will compare brain activation during the production of sentences in conditions that do or do not involve a competition for subjecthood and hence differ in their demand on functional level processing.

Among the few neuroimaging studies Haller, Radue, Erb, Grodd, Kircher [18] investigated the production of sentences by presenting their participants a list of incomplete stimuli (throw, ball, child) and asking them to produce Subject-Verb-Object sentences (“The child throws the ball”). The main result of the study was an activation in Broca's area (B44/45), consistent with many lesion studies reporting agrammatic syndrome in patients with B44/45 damage. The same conclusions were reached also by Dogil, Ackermann, Grodd, Haider, Kamp, et al. [19]. Participants produced German sentences upon the presentation of a word list. There was activation in Broca's and Wernicke's area suggesting that, in parallel to what is observed in agrammatic patients, syntactic processing involve Broca's area but also more complex neural circuits. Complex activation patterns were also observed by Kaan and Swaab [20] who, in addition to B44/45, observed activation also in the temporal network. Heim, Opitz, Friederici [21] also observed activation in area B44 in syntactic tasks requiring either word categorization or gender decision. Indefrey, Hagoort, Herzog, Seitz, Brown, [22] investigated the cortical activations of syntactic encoding in a positron emission tomography experiment. Participants were presented with scenes they had to describe by producing sentences of different complexity. In one condition they produced a full sentence (*the red square launches the blue ellipse*), in a further condition they produced just a sequence of noun phrases with a local syntactic structure but not a sentence level syntactic structure (*red square, blue ellipse, launch*), and in a third condition they just produced a list of single words with no syntactic relationship (*red, square, blue, ellipse, launch*). Relative to the baseline conditions, the full sentence condition elicited the activation of the left anterior rolandic operculum which varied with the complexity of the syntactic processing. Golestani [23] investigated the complexity of syntactic processing in the first (L1) and second (L2) languages of non-proficient late bilinguals. Participants either covertly read words or produced sentences from them. Sentence production activated Broca's area and supplementary motor area. Interestingly, the analyses performed on the LIFG revealed greater activation for L2 compared to L1, as a result of a neural activity for representations requiring an increased, more general, cognitive effort. Indeed, syntactic processing involves memory load capacity, attentional demands and executive processing [23]. Tettamanti and Weniger [24] pointed out that Broca's area may serve different cognitive functions, on the basis of fine-grained cytoarchitectonic parcellations and connections with different neural circuits. According to these authors, it is possible that Broca's areas support unspecified abstract hierarchical processes common to both language and other cognitive skills [25], [26].

In the present fMRI experiment we aimed to map the functional and positional level of grammatical encoding. Animate nouns are likely to be assigned the grammatical function of subject. We hypothesize that the production of a simple subject-verb-object sentence with two animate nouns introduces a competition for subjecthood or a competition at the functional level of processing. We expect that a similar sentence with an animate and inanimate noun does not induce such a competition.

In the functional level condition, participants were visually presented with three words (to chase, cat, mouse) and required to covertly produce a non reversible, semantically plausible sentence (the cat chases the mouse). In the positional level condition, the same materials were used except for the direct object that was inanimate (to chase, cat, ball). Also in this case participants were asked to covertly produce a non reversible, semantically plausible sentence (the cat chases the ball). To isolate regions involved in covert sentence production, a covert word reading task was introduced. Participants were required to covertly read the words visually presented. By comparing the functional and the positional level conditions we mapped the regions of activation of the functional level; where words are retrieved and the competition between two animate entities takes place. By comparing positional and word reading conditions we mapped the areas involved at the positional level of processing, where no competition is required, but where speakers still have to build the constituent structure and the correct word order.

## Material and Methods

### Ethics Statement

The Ethic Committee of the Ghent University Hospital approved the research. Participants gave written informed consent according to the institutional guidelines of the Ethic Committee of the Ghent University Hospital.

### Participants

Twenty healthy participants (six males) between 19 and 30 years old (mean ± SD: 23.2±3.3 years) took part in the study. All participants were native Dutch speakers with a normal or corrected-to-normal vision and right-handed as assessed by a handedness inventory [27]. None of the volunteers had a history of dyslexia or any neurological or psychiatric disorders.

### Procedure

Forty pairs of triplets (one verb and two nouns) were built. Each pair of triplets included a verb in the infinitive form (e.g. achtervolgen/to chase) and an animate noun (e.g. leeuw/lion). The triplets differed in the second noun chosen so that in one case it represented an animate entity (zebra/zebra) whereas in the other case it represented an inanimate entity (auto/car), resulting in two paired triplets (*achtervolgen*/to chase, *leeuw*/lion, *zebra*/zebra *vs achtervolgen*/to chase, *leeuw*/lion, *auto*/car).

Verbs were always put at the beginning of the triplets to avoid any biased order for the two nouns. The position of the nouns in the triplets was balanced. Triplets were built so that there was always one noun that could be a much more plausible agent than the other (e.g. it is semantically more plausible that a lion chases a zebra). Two tasks were chosen: in one task participants had to covertly produce a sentence (de *leeuw achtervolgt een zebra*/the lion chases the zebra), given the triplets (*achtervolgen, leew, zebra*); in the other they only had to covertly read the triplets of words (*achtervolgen, leeuw, zebra*). This resulted in a 2×2 factorial design with level of processing: functional (FUN) or positional (POS) and task: reading (READ) or sentence generation (SEN) as factors. Four different lists of four blocks each were built. Each block included ten triplets per condition. Before running the experiment, an independent sample of twenty participants performed the task out loud to verify that the triplets did not elicit passive sentences that could introduce additional confounding syntactic complexity. The results showed that participants produced active forms for 97% of the triplets. Frequency of occurrence for the nouns in the two conditions (zebra vs. auto) was calculated by means of the SUBTLEX-NL [28]. No significant difference was observed (F(1,39) = 2.35, p<0.13).

Each trial started with the presentation of the three words next to each other in black font Verdana size 20 in the centre of a white screen. To avoid a potential working memory load, the stimulus was displayed for 7 seconds. In order to separate the signal of consecutive stimuli, each stimulus was followed by a blank with a variable duration sampled from a skewed distribution (2, 3 or 5 seconds in respectively 54%, 31% and 15% of the trials). At the beginning of the experiment participants were instructed about the tasks they had to perform.

In order to inform the participants about the task to execute, each block either started with the instruction “read words” or with the instruction “produce sentence”, displayed on screen for 7 seconds. Within blocks, the word triplets in the functional and positional level conditions were intermixed and the order of the blocks and lists was counterbalanced across subjects. Both tasks were performed in silence to avoid any speech related movement artefacts. To build in a low-level measure of task performance and alertness, participants pressed a button when finishing the appropriate task for each trial.

Finally, several studies have demonstrated different activation patterns in human visual cortex for animate and inanimate objects [29]. Because the stimuli for the functional and positional level of processing only differ in the animacy of one word, obtained differences in brain activation could be fully attributed to similar category effects. To identify the brain areas involved in these potential category effects, subjects performed a localizer task after the 4 experimental runs. During this task, subjects were required to silently read the animate and inanimate nouns used in the stimuli for the main experiment. The 80 nouns were presented one by one for 0.5 seconds. The trials were separated by a blank of variable duration with the same timing parameters as the main experiment. Furthermore, by analogy with the main experiment, the trials were also randomly intermixed across two blocks and each block started with the instruction “read words”.

Stimulus presentation and response collection across the entire study was controlled using E-Prime (www.pstnet.com).

### Scanning procedure

The images were collected at 3T on a Magnetom TRIO MR scanner (Siemens Medical Systems, Erlangen, Germany) with an eight-channel PA head coil for radio-frequency transmission and signal reception. Whole brain functional images were acquired using a T2*-weighted sequence sensitive to BOLD contrast; 664 for the main experiment and 107 for the localizer task (EPI: TR = 2630 ms, TE = 35 ms, 40 axial slices, image matrix  = 64×64, FOV = 224 mm, flip angle  = 80°, voxel size  = 3.5×3.5×3.5 mm). A 3-D high-resolution T1-anatomical image of the whole brain was also obtained for coregistration with the functional images (3-D MPRAGE: TR  = 1550 ms, TE  = 2.39 ms, TI  = 900 ms, 176 sagittal slices, acquisition matrix  = 256×256, FOV  = 220 mm, flip angle  = 9°, voxel size  = 0.9×0.9×0.9 mm).

### Image analysis

The neuroimaging data were analysed using statistical parametric mapping (SPM5) (www.fil.ion.ucl.ac.uk/spm). The first four volumes of all EPI series were omitted from the analysis to allow the magnetisation to approach a dynamic equilibrium. First, the slices of each functional image were temporally realigned with the acquisition time of the middle slice. Next, motion artefacts were removed from the functional images by realigning all images to the mean image using a rigid body spatial transformation [30]. Using an affine transformation followed by a nonlinear transformation, the realigned functional images were normalized to a standard EPI template in the Montreal Neurological Institute stereotaxic space and re-sampled at an isotropic voxel size of 2 mm. Finally, the normalized images were smoothed with an isotropic 8-mm FWHM Gaussian kernel.

In the statistical model of the main experiment, the four conditions were each modelled separately using an event-related design. In order to detect potential time differences between conditions, the hemodynamic response for each event was modelled by means of a finite impulse response function using 6 time bins. The length of a time bin equalled one TR (2.63 sec), hence the finite impulse response model encompassed a total period of 15.78 sec, time locked to the start of each trial. This resulted in a total of 24 vectors that formed the covariates of interest in a general linear model [31]. The instruction at the start of each block was modelled in a similar way and the ensuing regressors were included in the statistical model as covariates of no interest, together with movement-related effects, low-frequency signal drifts over time and overall differences between sessions. The beta weights for all the covariates in the model were estimated by a least squares fit to the data.

For each participant we calculated linear contrasts of the corresponding beta weights for each time bin, averaged across sessions. This resulted in one SPM(t)-map per time bin for each contrast of interest. These SPM(t)s were then passed on to the second-stage analysis and modelled as a one-way ANOVA with time bin as factor and treating participants as a random variable [32]. To address the multiple comparisons problem resulting from the test calculated at each voxel, we used a minimum cluster size of 66 voxels to obtain a corrected extent threshold of p<0.05 at the cluster level (voxel level p<0.001, uncorrected) as was computed with Monte Carlo simulations (http://afni.nimh.nih.gov/pub/dist/doc/program_help/3dClustSim.html). For each subject, the final SPM(t)s were superimposed on the high-resolution anatomical scan in order to identify the corresponding anatomical regions of significantly activated clusters, and the anatomical details were compared with the atlas of [33].

A number of contrasts of interest were calculated. First, we identified the sentence generation network, irrespective of the level of processing ([FUN_SEN_ + POS_SEN_]>[FUN_READ_ + POS_READ_]). To ensure that the identified clusters at the second-stage analysis displayed a signal compatible with the hemodynamic character of the BOLD measure, the contrast weights were selected to reflect a canonical hemodynamic function subsampled at each time bin.

Second, we used the resulting group SPM(t) associated with the sentence generation network as a refined search space to detect potential differences between the two sentence generation tasks ([FUN_SEN_≠ POS_SEN_] inclusively masked with [FUN_SEN_ + POS_SEN_]>[FUN_READ_ + POS_READ_]). As we had no a priori hypothesis, we used an F-test to identify potential differences at any of the time bins. To further gain insight in these differences, we conducted a time course analysis using MarsBar [34]. First, we extracted the mean time course activity across the obtained clusters for FUN_SEN_ and POS_SEN_ in each time bin. Next, the per cent signal change was calculated and plotted per time bin, resulting in a peristimulus time histogram. A paired t-test was then computed for each time bin to assess the statistical significance of the potential differences between FUN_SEN_ and POS_SEN_.

Third, to exclude that the potential differences between FUN_SEN_ and POS_SEN_ may be attributed to category effects (since the stimuli only differ in the animacy of one noun), we included the data from a localizer task. In this task subjects read the animate and inanimate nouns used in the main experiment. In the statistical model, the start of each trial was convolved with a canonical hemodynamic response function to account for the hemodynamic signal in the data. The rest of the procedure was similar to the construction of the statistical model of the main experiment, resulting in two covariates of interest, animate and inanimate nouns. For each subject we calculated the contrast animate>inanimate and these were combined in a random effects group SPM(t) by means of a one-sample t-test. Finally, this obtained SPM(t) was used as an inclusive mask (threshold p<0.05, uncorrected) in the contrast described above to detect potential differences between FUN_SEN_ and POS_SEN_.

Furthermore, we tested if either of the sentence generation tasks recruited specific additional areas beyond the sentence generation network, by calculating the following contrasts in the voxels not included in the sentence generation network: [FUN_SEN_>POS_SEN_] inclusively masked with [FUN_SEN_>FUN_READ_], and [POS_SEN_> FUN_SEN_] inclusively masked with [POS_SEN_> POS_READ_]. Again, the contrast weights reflected a canonical hemodynamic function sub-sampled at each time bin.

## Results

**Figure 1:**
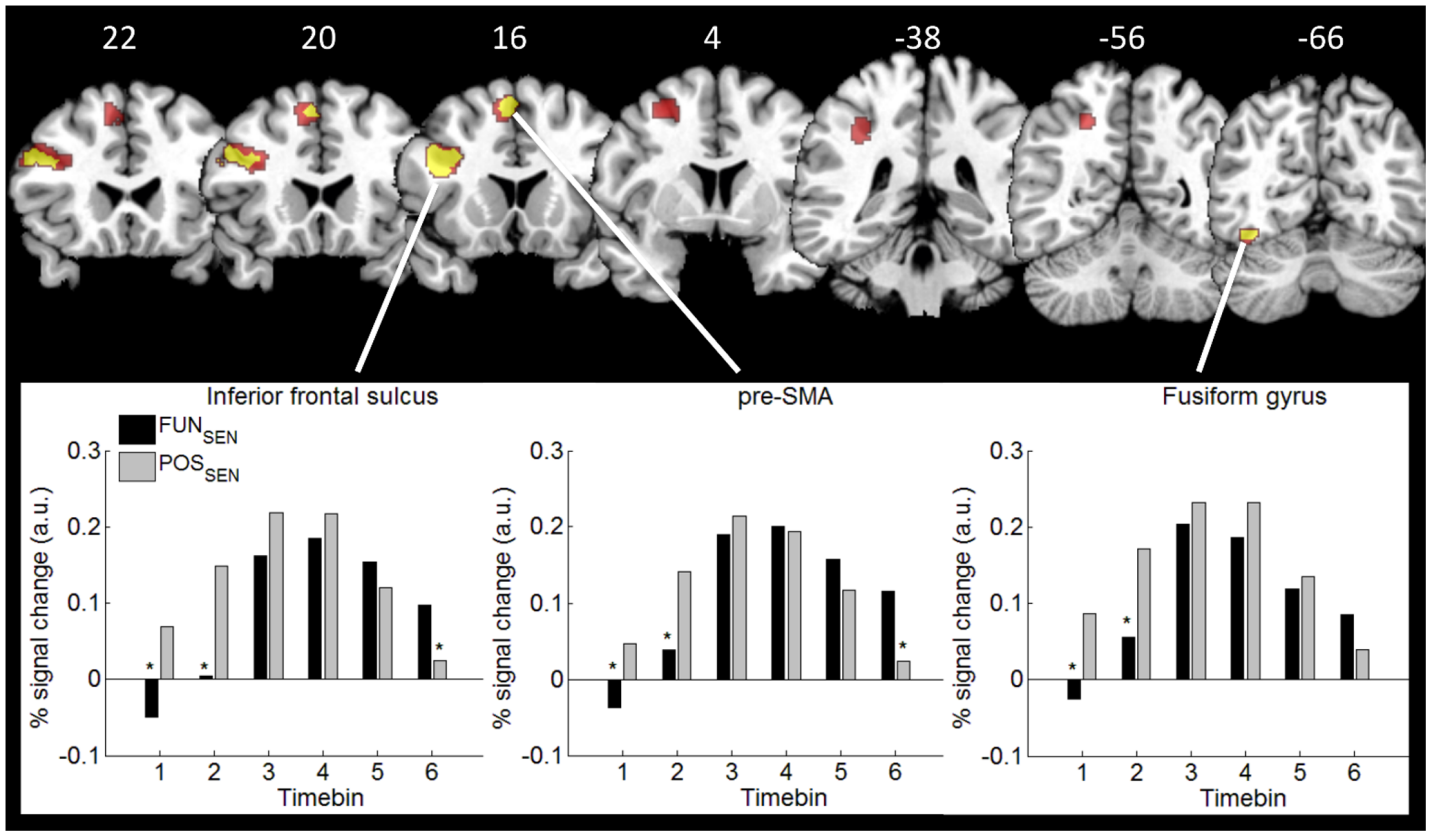
Sentence generation activation. An overview of common (red) and differential (yellow) covert sentence generation related activation, presented on a T1 weighted MNI single subject template (Eickhoff et al., 2005). The peristimulus time histograms summarize the results of the time course analysis across a period of approximately 16 sec time locked to the start of each trial. In three left hemisphere regions - inferior frontal sulcus, the VWFA and pre-SMA - the hemodynamic response associated with sentence generation at the functional level (FUN_SEN_) seems to set off later than sentence generation at the positional level (POS_SEN_) (* p<0.01).

Common sentence generation related activity was observed in a left lateralized network with significant clusters in the inferior frontal sulcus extending into the inferior frontal gyrus, the superior frontal sulcus, pre-SMA, anterior and middle IPS, and fusiform gyrus (see Table 1, Figure 1). The location of this latter cluster corresponds with the visual word form area or VWFA [35].

**Table 1:** Common sentence generation related activity: the sentence generation network.

Anatomical Region	Cluster size	Hemisphere	Z Score	Stereotaxic Coordinates		
[FUN_SEN_ + POS_SEN_]>[FUN_READ_ + POS_READ_]						
Inferior frontal sulcus	458	L	4.49	−38	16	24
Inferior frontal gyrus (p. triangularis)		L	3.41	−56	22	26
Superior frontal sulcus	158	L	4.24	−28	4	50
Middle IPS	107	L	3.99	−24	−56	44
pre-SMA	168	L	3.98	−10	20	48
Fusiform gyrus (VWFA)	86	L	3.97	−38	−66	−16
Anterior IPS	82	L	3.48	−34	−38	38

Within this network, three clusters showed differential sentence generation related activity (see Table 2). In the cluster in the inferior frontal sulcus that extends into the inferior frontal gyrus, the VWFA and pre-SMA the per cent signal change analysis reveals clear onset differences (Figure 1). Paired t-tests reveal that the hemodynamic response associated with sentence generation at the functional level seems to set off later than sentence generation at the positional level (VWFA: bin 1t(19) = −8.04 p<0.001, bin 2t(19) = −4.51 p<0.001; Pre-SMA: bin 1 t(19) = −3.43 p = 0.003, bin 2 t(19) = −3.84 p = 0.001, bin 6 t(19) = 4.68 p<0.001; inferior frontal sulcus: bin 1 t(19) = −4.98 p<0.001, bin 2 t(19) = −4.72 p<0.001, bin 6 t(19) = 2.91 p = 0.009). None of the areas showing differential sentence generation related activity displayed significant differences between reading animate and inanimate nouns, as revealed by the localizer data.

**Table 2:** Differential sentence generation related activity within the sentence generation network.

Anatomical Region	Cluster size	Hemisphere	Z Score	Stereotaxic Coordinates		
(FUN_SEN_ ≠ POS_SEN_) ∩ ([FUN_SEN_ + POS_SEN_]>[FUN_READ_ + POS_READ_])						
Fusiform gyrus(VWFA)	72	L	5.77	−38	−66	−14
pre-SMA	97	L	5.00	−6	10	54
Inferior frontal sulcus	369	L	4.99	−36	10	26
Inferior frontal gyrus		L	4.01	−54	20	22

Finally, only sentence generation at the functional level compared to positional level recruited extra areas that did not belong to the sentence generation network. This type of sentence generation additionally recruited bilateral precuneus (right: −6–66 32; left: 12–62 30; 423 voxels). Positional sentence generation compared to functional sentence generation did not recruit any extra areas not belonging to the sentence generation network.

## Discussion

Our data confirm that sentence generation in general requires neural circuits that several studies have indicated as responsible for syntactic processing [36]. Regions of activation for sentence generation have been found in the inferior frontal gyrus, inferior and superior frontal sulcus (BA44/45) of the left hemisphere. These areas have been often reported as responsible for semantic and syntactic processing [18]; [22] Haller et al. [18] observed a significant BOLD signal change in BA45 in a sentence generation task very similar to that of this study and they concluded that the network identified is responsible for syntactic encoding. In addition, in a scene description task reported by [22] in which participants had to produce sentences upon the visual presentation of objects “The red square launches the blue ellipse” the authors observed an activation of BA44. Interestingly, as Haller et al. [18] reported, the sets of activated areas of the two studies overlap strongly, suggesting that the activation in the left IFG may be better attributed to syntactic processing due to the restricted semantic processing required by the task of Indefrey et al. [22].

In a paper by Friederici et al. [37] the authors varied the semantics and the syntax in a sentence comprehension task and found activation in BA44 when the syntax of a sentence had to be processed even if single words were substituted by pseudowords. Even if in our study we examined production, our results suggest once again an involvement of Broca's areas in syntactic processing.

We also observed activation of the visual word form area (VWFA). Traditionally, this area is taken to support the activity connected with the initial word recognition stages during reading. However, Price et al. [38] demonstrated that VWFA is activated by a number of tasks that do not require visual word form processing such as naming pictures and colours, repeating auditory presented stimuli and reading Braille. Our results suggest that reading processes may be responsible for the activation of the VWFA which has been found active in the experimental conditions that required building a sentence, but also when the reading condition was presented to participants.

Activation of the pre-SMA is in general attributed to motor sequence control. However, only in recent years this area has been the object of intense scrutiny and the results provide evidence that it may be involved in several processes, from motor control to eye movements [39], and language processes such as making semantic decisions and word generation [40,41]. Cunnington, Windischberger, Deeke, Moser [42] and Nachev, Kennard, Husain [39] observed an activation of the pre-SMA for self-initiated compared to externally triggered movements. One possible hypothesis is that a covert sentence production task requires self-initiated internal activity, reflected in pre-SMA activation. Activations in the pre-SMA region have been previously observed in covert word production by Menenti et al. [43].

A difference in the hemodynamic activation between functional and positional level of processing was observed within the sentence generation network. Pre- SMA, VWFA, and the inferior frontal sulcus extending into the inferior frontal gyrus showed a delayed hemodynamic response onset in sentence generation at the functional level with respect to the positional level. None of these brain areas were involved in categorizing nouns as animate or inanimate, ruling out a possible semantic effect. In addition, one may argue that the competition effect observed originates during conceptualization, so that in the triplets chase/lion/zebra “lion” and “zebra” would be in competition to become the agent, hence there would be competition to conceptualise the event as a lion chasing a zebra or a zebra chasing a lion. However, as already described in the material section, the verb and the two nouns were selected to form a non-reversible sentence. As the triplets were paired and functional level condition differed from positional level only because of the two animate nouns, our interpretation is that the delay observed in areas crucial for production is given by the competition for subject position. Behavioural data seem to support this view. Since Bock and Warren [12], the hypothesis of conceptual accessibility has been taken to explain many of the effects found to describe the role of animacy in understanding grammatical encoding [44,45,46]. A concept which is more accessible for being animate, concrete or prototypical, or predicable, speeds up sentence generation because it is more rapidly assigned to the position of subject in an active sentence. This would explain why the brain activity in the positional level condition has an earlier onset in our study. In contrast, when two animate concepts have to be assigned to the role of subject in a sentence, the competition takes time thus resulting in a later onset of the activation pattern.

Though Broca's area has been associated with competition in sentence processing [47] the competition for subjecthood in our study did not increase activation in Broca's area. We found no significant differences in activation strength between the functional and positional sentence generation in Broca's area. This could be explained by a ceiling effect as sentence generation in general recruited Broca's area. Another possibility is that this competition is resolved by a different brain region, before the information is passed on to Broca's area. The additional recruitment of bilateral precuneus in the functional level condition further corroborates this view. Both attention allocation and processing of agency have been attributed to precuneus [48]. Resolving the conflict of assigning the role of subject might increase the attentional demand. Furthermore, a recent study suggests that the precuneus is involved in causal attributions in social situations [49]. It is possible that the activation of the precuneus in the functional level condition reflects the competition made by participants to assign the correct element to subject position.

Some behavioural data on syntactic priming effects challenged the idea that grammatical encoding consists of a series of different processes each devoted to the elaboration of a specific syntactic sub-process. For example, Pickering, Branigan, Mclean, [50] investigated syntactic priming in the dative structure. The sentence “*the driver showed the overalls with the stains to the mechanics*” (non-shifted form) has a grammatical alternative expressed in “*the driver showed to the mechanics the overall with the stains*” (shifted form). The two forms share the same relations but differ in how the constituents are ordered. On a two-stage view, the shifted version should have primed the non-shifted version. However, no such priming occurred and the authors concluded that dominance relations (functional level) and linear relations (positional level) are determined in a single stage. The results obtained here seem to contradict this view, rather suggesting that different neural circuits are involved at different stages of sentence production. To conclude, in an fMRI experiment we investigated the functional and positional level of processing. Though it is only a first step inside the syntactic box, the results provide some insights that may contribute to identify the neural paths supporting sentence production.
